# Patient and caregiver involvement in implementing health research in chronic kidney disease: a workshop report

**DOI:** 10.1093/ckj/sfaf021

**Published:** 2025-01-24

**Authors:** Dale Coghlan, Allison Jaure, Anastasia Hughes, Rebecca Wu, Andrea K Viecelli, Noa Amir, Hayley Candler, Brydee Cashmore, Yeoungjee Cho, Jonathan C Craig, Rosanna Cazzolli, Chandana Guha, Carmel M Hawley, Amandi Hiyare-Hewage, Martin Howell, Shilpanjali Jesudason, David W Johnson, Dominic Keuskamp, Feruza Kholmurodova, Karine Manera, Jasmin Mazis, Stephen McDonald, Shyamsundar Muthuramalingam, Javier Recabarren Silva, Amanda Sluiter, Armando Teixeira-Pinto, David J Tunnicliffe, Anita van Zwieten, Pushparaj Velayudham, Germaine Wong, Nicole Scholes-Robertson, the workshop investigators, Adela Yip, Alice Kennard, Allan Blackley, Allison Jaure, Amanda Dominello, Amanda Sluiter, Amandi Hiyare, Amber Williamson, Amy Luchterhand, Anastasia Hughes, Andrea Viecelli, Andrew Currey, Angela Rejuso, Anh Kieu, Anh Tho Tien, Anita van Zwieten, Annie Conway, Armando Teixeira-Pinto, Bill, Breonny Robson, Brydee Johnson, Carmel Hawley, Chandana Guha, Clarissa Hompas, Dale Coghlan, Darling Rojas-Canales, David J Tunnicliffe, Dominic Keuskamp, Effie Johns, Erandi Hewawasam, Felicity Lord, Fiona Carlisle, Fleur Tuthill, Germaine Wong, Gorjana Radisic, Hayley Candler, Heather Shepherd, Helen Coolican, Henry Pleass, Ieyesha Roberts, Irene Mewburn, Isabelle Haklar, Janelle Colquhoun, Janet Kelly, Jasmin Mazis, Javier Recabarren, Jill Brown, Joy Roberts, Karine Manera, Kathy Strudwick, Kim Nguyen, Luca Torrisi, Madeleine Parker, Madeleine Rapisardi, Martin Howell, Michael Collins, Natalie Brown, Nicky Boulter, Nicole Scholes-Robertson, Noa Amir, Pushparaj Velayudham, Rachel Li, Richard Le Leu, Rick Rapana, Robert Dale, Rosanna Cazzolli, Sadia Jahn, Sally Spence, Seethalakshmi Viswanathan, Shyamsundar Muthuramalingam, Yeoungjee Cho, Verlencia En ying chu, Victoria Sinka, Vishal Diwan, Wendy Hoy

**Affiliations:** Flinders University, Bedford Park, South Australia, Australia; Sydney School of Public Health, The University of Sydney, Australia; Centre for Kidney Research, The Children's Hospital at Westmead, Australia; Sydney School of Public Health, The University of Sydney, Australia; Centre for Kidney Research, The Children's Hospital at Westmead, Australia; Sydney School of Public Health, The University of Sydney, Australia; Centre for Kidney Research, The Children's Hospital at Westmead, Australia; Australasian Kidney Trials Network (AKTN), Centre for Health Services Research, Faculty of Medicine, The University of Queensland, Brisbane, Queensland, Australia; Department of Kidney and Transplant Services, Princess Alexandra Hospital, Brisbane, Queensland, Australia; Faculty of Medicine, The University of Queensland, Brisbane, Australia; Sydney School of Public Health, The University of Sydney, Australia; Centre for Kidney Research, The Children's Hospital at Westmead, Australia; Australasian Kidney Trials Network (AKTN), Centre for Health Services Research, Faculty of Medicine, The University of Queensland, Brisbane, Queensland, Australia; Sydney School of Public Health, The University of Sydney, Australia; Centre for Kidney Research, The Children's Hospital at Westmead, Australia; Australasian Kidney Trials Network (AKTN), Centre for Health Services Research, Faculty of Medicine, The University of Queensland, Brisbane, Queensland, Australia; Department of Kidney and Transplant Services, Princess Alexandra Hospital, Brisbane, Queensland, Australia; Faculty of Medicine, The University of Queensland, Brisbane, Australia; Flinders University, Bedford Park, South Australia, Australia; Sydney School of Public Health, The University of Sydney, Australia; Centre for Kidney Research, The Children's Hospital at Westmead, Australia; Sydney School of Public Health, The University of Sydney, Australia; Centre for Kidney Research, The Children's Hospital at Westmead, Australia; Australasian Kidney Trials Network (AKTN), Centre for Health Services Research, Faculty of Medicine, The University of Queensland, Brisbane, Queensland, Australia; Department of Kidney and Transplant Services, Princess Alexandra Hospital, Brisbane, Queensland, Australia; Faculty of Medicine, The University of Queensland, Brisbane, Australia; Flinders University, Bedford Park, South Australia, Australia; Sydney School of Public Health, The University of Sydney, Australia; Centre for Kidney Research, The Children's Hospital at Westmead, Australia; Menzies Centre for Health Policy and Economics, The University of Sydney, Sydney, NSW, Australia; Faculty of Health and Medical Sciences, University of Adelaide, Adelaide, South Australia, Australia; Central and Northern Adelaide Renal and Transplantation Service, Royal Adelaide Hospital, Adelaide, South Australia, Australia; Australasian Kidney Trials Network (AKTN), Centre for Health Services Research, Faculty of Medicine, The University of Queensland, Brisbane, Queensland, Australia; Department of Kidney and Transplant Services, Princess Alexandra Hospital, Brisbane, Queensland, Australia; Faculty of Medicine, The University of Queensland, Brisbane, Australia; Faculty of Health and Medical Sciences, University of Adelaide, Adelaide, South Australia, Australia; Australia and New Zealand Dialysis and Transplant Registry (ANZDATA), South Australian Health & Medical Research Institute (SAHMRI), Adelaide, South Australia, Australia; Australia and New Zealand Dialysis and Transplant Registry (ANZDATA), South Australian Health & Medical Research Institute (SAHMRI), Adelaide, South Australia, Australia; Sydney School of Public Health, The University of Sydney, Australia; Centre for Kidney Research, The Children's Hospital at Westmead, Australia; Australia and New Zealand Dialysis and Transplant Registry (ANZDATA), South Australian Health & Medical Research Institute (SAHMRI), Adelaide, South Australia, Australia; Faculty of Health and Medical Sciences, University of Adelaide, Adelaide, South Australia, Australia; Central and Northern Adelaide Renal and Transplantation Service, Royal Adelaide Hospital, Adelaide, South Australia, Australia; Australia and New Zealand Dialysis and Transplant Registry (ANZDATA), South Australian Health & Medical Research Institute (SAHMRI), Adelaide, South Australia, Australia; Central Adelaide Local Health Network, Adelaide, South Australia, Australia; Sydney School of Public Health, The University of Sydney, Australia; Centre for Kidney Research, The Children's Hospital at Westmead, Australia; Sydney School of Public Health, The University of Sydney, Australia; Centre for Kidney Research, The Children's Hospital at Westmead, Australia; Sydney School of Public Health, The University of Sydney, Australia; Centre for Kidney Research, The Children's Hospital at Westmead, Australia; Sydney School of Public Health, The University of Sydney, Australia; Centre for Kidney Research, The Children's Hospital at Westmead, Australia; Sydney School of Public Health, The University of Sydney, Australia; Centre for Kidney Research, The Children's Hospital at Westmead, Australia; Australasian Kidney Trials Network (AKTN), Centre for Health Services Research, Faculty of Medicine, The University of Queensland, Brisbane, Queensland, Australia; Sydney School of Public Health, The University of Sydney, Australia; Centre for Kidney Research, The Children's Hospital at Westmead, Australia; Sydney School of Public Health, The University of Sydney, Australia; Centre for Kidney Research, The Children's Hospital at Westmead, Australia

To the Editor,

Patient and caregiver (consumer) involvement in research can strengthen the relevance and uptake of evidence [1, 2]. However, there is little guidance on how to involve consumers in the implementation of research in practice and policy [3, 4]. Existing frameworks suggest that consumers can contribute to implementation of research by providing input on the design and delivery of interventions to ensure acceptability in practice [1]. Consumers can also contribute to strategies for implementing research, for example by identifying potential barriers and facilitators to adopting new treatments or providing feedback on implementing interventions through surveys or interviews [3, 5—7]. In the context of chronic kidney disease (CKD), there is little documented on consumer involvement in the implementation of research. The aim of this workshop was to identify ways to involve patients and caregivers in the implementation of research in CKD.

We convened a workshop involving patients with CKD and their caregivers (*n* = 27), and health professionals (*n* = 54) with simultaneous workshops in Adelaide, Brisbane, Sydney (in person), and online. Across 10 facilitated breakout groups using set questions, participants discussed approaches to involve consumers in the implementation of research. Further details are available in the Supplementary File.

We identified two themes that reflected strategies for patient and caregiver involvement in the implementation of research in CKD: building engagement and familiarity, and harnessing the voices of consumers in advocacy. The themes and subthemes are described in the following sections, and illustrative quotations are provided in Supplementary Item S1. Fig. 1 depicts the relationship among the themes.

**Figure 1:**
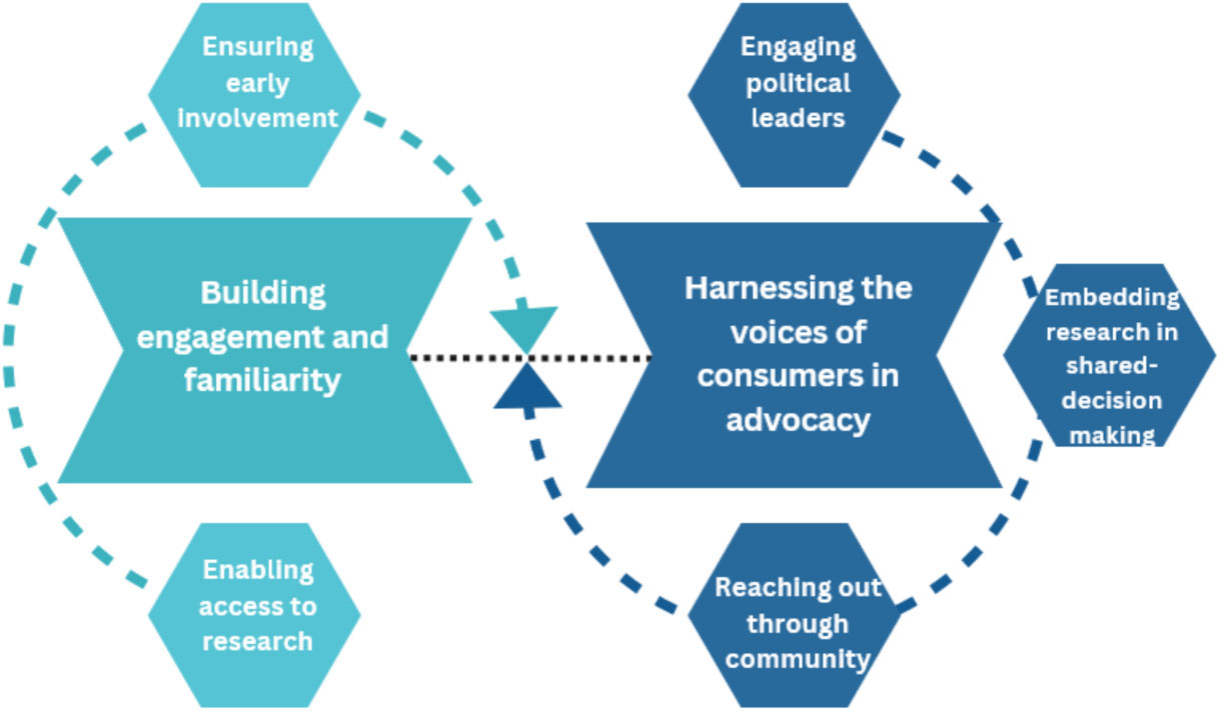
Strategies for patient and caregiver involvement in the implementation of CKD research: a thematic schema.

## ENSURING EARLY INVOLVEMENT

Participants emphasized that the involvement of consumers at the earliest stages of the research (e.g. setting research priorities and developing the research questions) would empower consumers with a ‘greater voice’ to meaningfully contribute to the research output and direction. Consumers stated that a ‘true partnership’ formed ‘from the very get-go’ of the study would motivate them to help implement research, as such studies are more likely to be relevant and meaningful to them. This approach fosters trust and reciprocity with researchers and the product of their work, ensuring research implementation aligns with consumer priorities.

## ENABLING ACCESS TO RESEARCH

Consumers needed convenient access to research to support its implementation. Consumers often conveyed that they were unaware of what research was being undertaken and without their meaningful involvement they could not implement the findings into practice, for example, in their own health decision making.

## EMBEDDING RESEARCH IN SHARED DECISION MAKING

Consumers suggested that they could have a direct role in implementing research in their own individual health decision making, or more broadly, in translating research evidence into decision-making tools. Consumers wanted to ‘actively participate’ and be ‘confident to voice’ their treatment preferences based on research findings, highlighting the need for research outputs to be ‘consumer friendly’ (i.e. plain language, ease of access) so they could understand the evidence to advocate for their needs in clinical care.

## REACHING OUT THROUGH COMMUNITY

Consumers emphasized the value of ‘peer sharing’ and ‘leveraging community networks’ to amplify advocacy efforts to support the implementation of research in CKD. They emphasized that receiving information from other patients who had been involved in research experience was valuable, as they could gain practical insights and advice to help understand research and to work together to develop a plan of action to drive research implementation. They felt that there was power in their collective voice, ultimately strengthening advocacy efforts for the implementation of research that they believed was valuable and important.

## ENGAGING POLITICAL LEADERS

Advocating at the political level through collective lobbying was emphasized as a way to implement research findings in practice and policy decisions. Consumers suggested that they could engage local politicians by ‘writing letters’ and ‘making appointments’, and advocate for implementing research findings that could improve patient-centred care and effectively change policy.

The two key strategies highlighted by consumers in this workshop included engaging consumers early in the research process to motivate and build confidence to be involved in implementing the findings, and involving consumers in advocacy efforts. Consumers expressed they are often unaware of research opportunities available, and their potential role and power to influence research implementation. Consumers felt they could be involved in the direct implementation of research in the context of their personal shared decision making in clinical care and influencing clinical care more broadly.

Frameworks for the implementation of research [5, 6, 8] similarly suggest that consumers should be involved in the initial stage of a research study to maximize implementation [7, 9]. The Medical Research Council framework also provides valuable guidance on integrating consumer input throughout the research process, including as early as formulating the research question.^S1^ Early and meaningful involvement of consumers not only clarifies objectives and ensures research relevance but also plays a critical role in identifying potential barriers to implementation through supporting designs of interventions and programmes likely to have greater acceptability and feasibility. Adopting this strategy can clarify objectives, ensure the research is meaningful and relevant to consumers, and foster their motivation and inclusion in the execution of implementation strategies.

There is a small amount of evidence on patient and caregiver involvement in the implementation of research in CKD. For example, patients with CKD were involved in an eHealth service intervention for self-management to help ensure that the online tools and resources addressed their needs, leading to greater engagement, higher satisfaction, and more successful self-management outcomes.^S2^ In another study, patients with CKD were involved in the development and implementation of individualized care plans, which enhanced their motivation to actively participate in the treatment.^S3^ Ongoing feedback mechanisms were established during the implementation of their care plans, which ensured the continuous integration of their preferences and challenges in the plan, and ultimately fostered a greater sense of ownership and active adoption of their treatment plans.^S4^

Our workshop highlights that consumers desire to actively support the implementation of research. In the context of shared decision making, consumers may be able to implement research findings directly in their own care or contribute to the development of decision-making tools based on the evidence.

Moreover, consumers can individually or collectively advocate for the implementation of research findings by engaging policymakers, healthcare providers, and other key stakeholders to ensure that research is translated into practice. Consumer representatives lobbying for government support could influence and change policy and political agendas, demonstrating the power of advocacy in translating research into actionable outcomes.^S4^ Advocacy organizations, local community groups, and peer navigators possess trusted voices and relationships. They can serve as conduits and provide input to ensure research is translated into practice, such as in the development of patient tools and outreach programmes.^S5^

Further efforts are needed to involve consumers in the implementation of research in CKD. By building early engagement and familiarity, and harnessing consumers’ voices in advocacy, research can be more effectively translated into practice and policy, ultimately improving patient outcomes and healthcare.

**Table:** 

First name	Last name	Primary Affiliation	City	State
Adela	Yip	The University of Sydney	Sydney	NSW
Alice	Kennard	Canberra Health Services	Canberra	ACT
Allan	Blackley	Consumer—patient	Invercargill, New Zealand	
Allison	Jaure	The University of Sydney	Sydney	NSW
Amanda	Dominello	Consumer—The University of Sydney	Sydney	NSW
Amanda	Sluiter	Consumer—The University of Sydney	Sydney	NSW
Amandi	Hiyare	Flinders University	Adelaide	SA
Amber	Williamson	Consumer—patient	Brisbane	QLD
Amy	Luchterhand	Consumer—caregiver	Brisbane	QLD
Anastasia	Hughes	The University of Sydney	Sydney	NSW
Andrea	Viecelli	The University of Queensland	Brisbane	QLD
Andrew	Currey	Consumer—patient	Blue Mountains	NSW
Angela	Rejuso	The University of Sydney	Sydney	NSW
Anh	Kieu	The University of Sydney	Sydney	NSW
Anh Tho	Tien	Consumer—patient	Brisbane	QLD
Anita	van Zwieten	The University of Sydney	Sydney	NSW
Annie	Conway	Australian and New Zealand Dialysis and Transplant Registry	Adelaide	SA
Armando	Teixeira-Pinto	The University of Sydney	Sydney	NSW
Bill		HP	Adelaide	SA
Breonny	Robson	Kidney Health Australia	Adelaide	SA
Brydee	Johnson	The University of Sydney	Sydney	NSW
Carmel	Hawley	The University of Queensland	Brisbane	QLD
Chandana	Guha	Consumer—caregiver—The University of Sydney	Sydney	NSW
Clarissa	Hompas	Australian and New Zealand Dialysis and Transplant Registry	Adelaide	SA
Dale	Coghlan	Flinders University	Adelaide	SA
Darling	Rojas-Canales	Flinders University	Adelaide	SA
David J.	Tunnicliffe	The University of Sydney	Sydney	NSW
Dominic	Keuskamp	Australian and New Zealand Dialysis and Transplant Registry	Adelaide	SA
Effie	Johns	Consumer—patient	Adelaide	SA
Erandi	Hewawasam	Australian and New Zealand Dialysis and Transplant Registry	Adelaide	SA
Felicity	Lord	Consumer—patient	Adelaide	SA
Fiona	Carlisle	Consumer—patient		NSW
Fleur	Tuthill	SA Health	Adelaide	SA
Germaine	Wong	The University of Sydney	Sydney	NSW
Gorjana	Radisic	Royal Adelaide Hospital	Adelaide	SA
Hayley	Candler	The University of Queensland	Brisbane	QLD
Heather	Shepherd	The University of Sydney	Sydney	NSW
Helen	Coolican	Consumer—PKD Foundation Australia	Sydney	NSW
Henry	Pleass	The University of Sydney	Sydney	NSW
Ieyesha	Roberts	Consumer—patient—The University of Sydney	Kempsey	NSW
Irene	Mewburn	Consumer—patient	Brisbane	QLD
Isabelle	Haklar	South Australian Health and Medical Research Institute	Adelaide	SA
Janelle	Colquhoun	Consumer—patient		QLD
Janet	Kelly	University of Adelaide	Adelaide	SA
Jasmin	Mazis	Australian and New Zealand Dialysis and Transplant Registry	Adelaide	SA
Javier	Recabarren	The University of Sydney	Sydney	NSW
Jill	Brown	Consumer—patient	Sydney	NSW
Joy	Roberts	Consumer—patient	Adelaide	SA
Karine	Manera	The University of Sydney	Sydney	NSW
Kathy	Strudwick	Consumer—patient	Adelaide	SA
Kim	Nguyen	HP		SA
Luca	Torrisi	Consumer—patient	Sydney	NSW
Madeleine	Parker	Consumer—patient	Sydney	NSW
Madeleine	Rapisardi	Consumer—patient	Sydney	NSW
Martin	Howell	The University of Sydney	Sydney	NSW
Michael	Collins	Royal Adelaide Hospital	Adelaide	SA
Natalie	Brown	Consumer—patient	New Zealand	
Nicky	Boulter	The University of Sydney	Sydney	NSW
Nicole	Scholes-Robertson	Consumer—The University of Sydney	Alice Springs	NT
Noa	Amir	The University of Sydney	Sydney	NSW
Pushparaj	Velayudham	The University of Queensland	Brisbane	QLD
Rachel	Li	The Royal Flying Doctor	Adelaide	SA
Richard	Le Leu	Central and Northern Adelaide Renal and Transplantation Services	Adelaide	SA
Rick	Rapana	Consumer—patient	New Zealand	
Robert	Dale	Consumer—patient	Adelaide	SA
Rosanna	Cazzolli	The University of Sydney	Sydney	NSW
Sadia	Jahn	University Health Network	Adelaide	SA
Sally	Spence	Consumer—patient	New Zealand	
Seethalakshmi	Viswanathan	ICPMR Westmead/University of Sydney	Sydney	NSW
Shyamsundar	Muthuramalingam	Consumer—patient	Adelaide	SA
Yeoungjee	Cho	The University of Queensland	Brisbane	QLD
Verlencia	En ying chu	OSCAR Care Group	Adelaide	SA
Victoria	Sinka	The University of Sydney	Sydney	NSW
Vishal	Diwan	The University of Queensland	Brisbane	QLD
Wendy	Hoy	Metro North Health	Brisbane	QLD

## Supplementary Material

sfaf021_Supplemental_File

## Notes

A complete list of the investigators is provided in the acknowledgements section
