# Supplementary material for: Patient and caregiver involvement in implementing health research in chronic kidney disease: a workshop report
Source: Clin Kidney J. 2025 Jan 24;18(3):sfaf021. doi: 10.1093/ckj/sfaf021 (PMC11880764; doi:10.1093/ckj/sfaf021)
Supplement: sfaf021_Supplemental_File [file sfaf021_supplemental_file.docx]

### **Item S1: Illustrative quotations**

| Theme | Quotation |
| --- | --- |
| Building Engagement and Familiarity | |
| Ensuring early involvement | - "If we don't go to the people with lived experience, we're missing one-half of that implementation story." (Consumer) - “True partnership with consumers [...] From the very get-go of the study design.” (Consumer) - “Obviously the further down the list we are involved [...] the less empowered we feel and the less valued we feel.” (Consumer) - “Involvement at that level [...] is what's giving us a greater voice on behalf of the consumers in our area.” (Consumer) |
| Enabling access to research | - "The barriers are […] firstly, around finding the research." (Consumer) - "We don't want our nephrologist to be the source of our research information. There should be other avenues…" (Consumer) - "Access to opportunities to be involved in implementation” – “Access to information which generates the knowledge”. (Health Professional) - "What we do as a group collectively makes a meaningful difference and really reaches everyone regardless of where you are, what ethnic origin you are, or what your disease modality is, ensuring that you have ability to access and be empowered in that journey." (Health Professional) - "Well, obviously, they've got to know what the research is to start with, and what the research is purporting to do." (Consumer) |
| Harnessing the consumer voice in advocacy. | |
| Embedding research in shared decision-making. | - "Forming those relationships with your clinician, where you feel confident enough to voice maybe a treatment path that are different or doing something different requires a certain amount of trust between the two…” (Consumer) - "Not just producing output that is targeted at the doctors and nurses and other healthcare professionals but making content that is actually available and accessible to people with lived experience, so they can go into those clinical appointments and have a shared decision about what's the best options for them with their doctors." (Health Professional) - "Shared Goals of Care: So, if anybody enters hospital here, they have their plan worked out with their medical team and agree to it. And so, it's allowing patients to have a say in what's done with them." (Consumer) - "We are hoping that involvement at the decision-making level, is what's giving us a greater voice on behalf of the consumers in our area." (Consumer) - "If consumers understand the results of trials and studies… they can then take that to their doctor and say, "I've heard this. Have you heard this? Can we try this?"…? (Consumer) |
| Reaching out through community | - “So, peer sharing is key... because that's someone with a lived experience with the same condition, same interests.” (Consumer) - "Connecting to those networks, using those network champions and maybe developing this implementation science approach with consumers and having a plan." (Consumer) - "The value of peer information and peer referral and how helpful it is to receive information from people who are in a similar situation as oneself." You have peers who are experienced and feel comfortable in the world of research, they may have a role in guiding other patients…” (Health Professional) |
| Engaging political leaders | - "Annoy your local politicians. And any other politician. Whether by writing letters, making appointments, going, and talking to people.” (Consumer) - "Advocate for the clinical care change and the policy change because it will be different right around the world and at different sites depending on the different levels of governments like you said”. (Health Professional) |
|  | |

### **Item S2: Supplementary Methods**

Context and overview: Better Evidence and Translation – Chronic Kidney Disease (BEAT-CKD) is a collaborative research network that aims to generate high-quality research evidence to improve outcomes for people with CKD. The government-funded network includes patients, caregivers, health professionals from CKD research, and platforms from across Australia and New Zealand.

Background: A workshop was conducted on patient and caregiver involvement in CKD research. In this report, we focused on the topic consumer involvement in research implementation discussed in part 2 of the workshop. Across ten facilitated breakout groups, participants discussed the set questions (outlined in Supplementary File 3) and were allocated 25 minutes for discussion. In total, 77 people from eight states/territories in Australia (Australian Capital Territory, New South Wales, the Northern Territory, Queensland, South Australia, Tasmania, Victoria, Western Australia), and four people from New Zealand contributed to the workshop.

Each facilitator was provided with a question guide and nominated a representative from their group to provide a summary of their discussion back to the plenary. Transcripts were entered into NVIVO for coding and analysis. Concepts relating to patient/caregiver involvement in the implementation of the research were identified and grouped into themes by the first author (DC), which were then reviewed by other facilitators and investigators (AJ, NS-R, AH, RW) to ensure that all data were captured into the themes. All participants received a draft workshop report to provide feedback, and additional comments were integrated and addressed in the final report.

### **Item S3: Facilitator question guide for breakout discussion.**

| **Implementation – how can consumers be involved in implementing research in practice and in policy?**   1. **How can consumers be involved in helping to ensure that research is applied in clinical care and policy?**     1. Prompts – discuss responsibility and roles, examples, advocacy 2. **What are some barriers or challenges for consumers, and what resources or support are needed for consumers to be involved in implementation?** |
| --- |

### **Item S4: Supplementary References**

S1. Shahsavari H, Matourypour P, Ghiyasvandian S, Nejad MRG. Medical Research Council framework for development and evaluation of complex interventions: A comprehensive guidance. *J Educ Health Promot*. 2020;9:88. doi:10.4103/jehp.jehp_649_19

S2. Donald M, Beanlands H, Straus S, et al. A Research Protocol for Implementation and Evaluation of a Patient-Focused eHealth Intervention for Chronic Kidney Disease. *Glob Implement Res Appl*. 2022;2(1):85-94. doi:10.1007/s43477-022-00038-3

S3. Dorough A, Forfang D, Mold JW, Kshirsagar AV, DeWalt DA, Flythe JE. A Person-Centered Interdisciplinary Plan-of-Care Program for Dialysis: Implementation and Preliminary Testing. *Kidney Med*. Mar-Apr 2021;3(2):193-205.e1. doi:10.1016/j.xkme.2020.11.010

S4. Neubeck L, Galbraith M, Drossart I, Mindham R. The essential role of patients in advocacy and policy. *European Heart Journal*. 2023;44(28):2506-2507. doi:10.1093/eurheartj/ehad312

S5. Luyckx VA, Tuttle KR, Abdellatif D, et al. Mind the Gap in Kidney Care: Translating What We Know Into What We do. *Can J Kidney Health Dis*. 2024;11:20543581241252506. doi:10.1177/20543581241252506
